# Transactions of the American Medical Association

**Published:** 1859-05

**Authors:** 


ART. IV.—*Transactions of the American Medical Association. Vol. XI.*

The volume before us contains the report of the Transactions of the American Medical Association, for its Eleventh Annual Meeting held in the city of Washington, D. C., May 4th, 1858.

It has been delayed somewhat beyond the usual time of publication, and for which delay the Committee of publication affix an apologetical note for the time consumed in the preparation of the volume, assigning as a cause, the delay in returning proofs that were sent to different points for correction. This has been the sole and entire cause for the non-appearance of the volume, at an earlier date. We have no heart to find fault with the committee of publication for the tardiness in the appearance of the volume. It must be a labor of love only, which could prompt a committee unpaid, to take charge of the publication of so ponderous a volume, and submit to the many vexations incidental to its preparation, the principle of which were beyond their control, and perhaps were from the carelessness, if not neglect of parties who expected to be glorified in this very volume. We only wonder they are enabled to present a book so unexceptional in its typographical arrangement and appearance, as is presented in this specimen of their painstaking labor. In passing, we will intrude the remark, that the publishing committee of the Transactions of our State Society might take several lessons from our Philadelphia brethren without any detriment, and to the manifest comfort of their readers.

The daily proceedings of the Association have already been noticed in the last June number of this Journal, and we shall not, therefore, occupy the present time in recapitulating the minutes of each day's session. There seemed to be about the ordinary amount of discussion upon those interminable subjects of debate, medical reform, medical education, medical literature, and alterations of the constitution. A little variety by way of spice, was thrown in, in the debate which ensued upon the apology of Dr. David M. Reese, for endorsing Prof. McClintock for an appointment to the Blockley Hospital, Philadelphia; and from an effort to dictate to the Secretary of the Treasury the proper person to be appointed as inspector of Drugs and Medicines for the port of New York. That apparently exhaustless subject of comment and communications, the gift of a stone to the Washington monument, occupies a couple of pages, to the edification of the writers of the correspondence upon the subject. We think it about time that "that stone" was fixed in the monument and disposed of, or some other plan arranged by which the profession of the land could be relieved from the annual recitation of the wonderful genius of the "young man, a native of Litiz, Pennsylvania, who had given unmistakable evidence of genius as a sculptor," and who, *singularly enough,* was in the employ of "a marble mason of the city of Lancaster, Pennsylvania;" and that five hundred of the one thousand dollars paid for the execution of the work by the Association, was devoted to the young artist then in Europe. (How many young physicians in Europe would dislike a similar gift from the Association?)

We notice that the New Jersey Medical Society presented a series of resolutions, the pith of which was recommending a new title, or degree, to be conferred by the Association upon its members, M. A. M. A., *Members of the American Medical Association;* and that a Board of Censors for each circuit of the United States Supreme Court, be appointed for the purpose of examining candidates for the title of "Member of the American Medical Association," on whose certificate the President of the Association, shall, after the candidate has subscribed to the Code of Ethics of the Association, and paid *blank* dollars into its treasury, issue a diploma, setting forth the fact of membership. We imagine the Association would have "a good time" in trying to carry any such regulation into execution. If all, or one-tenth part of the visionary projects for medical reform which have been proposed to the Association since its organization had been attempted to be carried out, its fate would have been sealed long ago. Fortunately a large majority of the propositions and amendments which are "laid over for one year, under the rules," are never heard of afterwards.

The Indiana State Medical Society is anxious for some system for the interchange of the *Transactions* of the various local societies of the United States, and invokes the aid of the Association in carrying out the project.

An examination of the contributions to the volume in connection with the daily proceedings of the Association, discloses some things which require an explanation upon the part of the committee of publication which they have not given. Either the secretaries have performed their duties very imperfectly, and given the Association a very meagre report of the daily proceedings, or else some sort of "hocus pocus" has been employed by somebody, by which papers which were never presented to the association are published among the reports, and one paper at least, which was presented to the Association, read and illustrated, and ordered published, does not appear in the Transactions.

To be explicit, the *Reports on Moral Insanity in its Relation to Medical Jurisprudence*, By Dr. Meredith Reese, of New York; *On Stomatitis*

*Materna*, By Dr. D. L. M'Gugin, of Keokuk; and on the *True Position and value of Operative Surgery as a Therapeutic Agent*, By Prof. J. B. Flint, of Louisville, find no mention in the daily proceedings as having ever been presented to the association, or any intimation that the association was ever aware that they had been prepared and presented.

Again, Dr. J. M. Sims, of New York, read a report on the *Treatment of the Results of Obstructed Labor*, which was accepted and referred to the committee on publication. Why was not Dr. Sims's report published? And why were these others published if they were never presented to the association?

It is scarcely to be presumed that these were voluntary communications, for although a large number of these are yearly presented by aspirants for the honor of having their name included in the list of contributors, and a committee is regularly appointed to receive them, we know that it has been the policy to discourage the presentation of voluntary communications, and to restrict the association to the regular reports of the committees which they appoint; claiming that they should have some knowledge of the capacities of the men who undertake to speak for the association in matters which so intimately affect their standing with the medical world; and the committee of reference serves only to furnish an honorable grave in which to inter the free will offerings of volunteers.

We think it would be a good plan, as it would save many a heart ache, if the association would distinctly announce that voluntary communications are not desired, except they be submitted for the competition of the prizes offered by the association.

Having said so much, by way of introduction, in which, perhaps, not a few will think we have managed to introduce no little fault finding, we address ourselve to the task, which at best must be but imperfectly performed, of examining the contents of the volume, and giving some expression as to the value and distinctive characters of the several papers of which it is composed. It is no easy task to compress within the compass which these pages will admit, a fitting notice of a volume of a thousand pages, made up of contributions upon almost all of the several departments of medicine and surgery. It is scarcely possible to do more than indicate the peculiar characteristics of the several reports and papers, indicating their authors, and leaving each reader to arrive at his own conclusions as to their real values. This, however, is scarcely to be expected from what purports to be a review of an important publication; and, to such as may not be able to command the perusal of the volume, or lack the industry necessary for its careful perusal, and at the same time feel desirous to talk learnedly of its contents, building themselves upon the foundations laid in the perusal of a review, may be the cause of serious complaint. We shall, however, endeavor as far as possible to indicate the character of this volume.

Before referring to any article of its contents, we shall indulge in one remark, that the present volume gives indisputable evidence that the zeal in behalf of the American Medical Association which characterized its organization and the early years of its existence, has suffered no diminution. The Association has now a strong hold upon the affections of the profession of the country, and it is looked to as a great conservative power in these days of professional degeneracy,and it remains for its members only to be true to the trusts which are delegated to them, and to cement as far as possible, and as firmly as possible, the different sections of our land in the behalf of legitimate medicine, to cause the fires of quackery to pale before the brighter radiance of the flames kindled upon the altars of true science.

Passing the Address of the *President*, Dr. Paul F. Eve, of Tennessee, which is a graceful recitation of what the association has accomplished for the advancement of medical science, we come to the first paper in the order of the arrangement of the volume,—*The Report on the Medical Topography of the Epidemic Diseases of Kentucky*, by W. L. Sutton, M. D.; occupying some ninety pages. The paper is an exceedingly interesting one, presenting many valuable facts in reference to the medical history of the State, and possessing merit far beyond many similar reports which have been made to the association. Dr. Sutton is a veteran in matters having reference to epidemic and sanitary questions. If he has not himself prepared, he has had charge of the Registration Reports of the State of Kentucky, for several years. He has a natural taste for such labors; and perfected by practice, he is enabled to give us a valuable paper upon the subject embraced within its range. He has accompanied his report with a map exhibiting the geological formations of the State as an additional means of illustration of the various causes to be taken into account among the question of the causations of disease.

Dr. Sutton's Report is followed by two others, upon kindred subjects:—*Report on the Topography and Epidemic Diseases of New Jersey, and the Treatment Thereof*, By Lyndon Smith, M. D.; and the *Report of the Committee on the Epidemics of Ohio*, By Geo. Mendenhall, M. D.

These two are exceedingly short reports, and serve principally to record the epidemic influences for the year or two past, and as such records are valuable to the sanitarian.

If the medical topography of America is never written, it is very evident it will be no fault of the profession as represented in the association, as any one must be convinced who will turn over the volumes of its Transactions. The great difficulty is, that a vast amount of imperfect and useless materials is collected together, and when one in search of the solution of some question of vital statistics, supposes he has but to turn to the garnered facts of these several volumes for an answer to his inquiries, he finds too often, upon search, that the imperfect character of the records and statistics renders perfectly valueless to him, the labors of the reporters, and the wonder to his mind is, who should take the pains to collect such imperfect data. We presume, however, that for the present this is unavoidable. We must accept what is given us, trusting for a larger experience to furnish for the future what the true wants of the profession demand. It is certain that from no other source, can our national medico-topographical history be so easily or so perfectly written as by the members, and from the contributions of the members of the American Medical Association.

The next paper is the *Report of the Committee on Medical Literature*, By Dr. A. B. Palmer, of Michigan. This is one of the stereotyped subjects of report, which are yearly presented, or expected to be presented, to the association. Prof. Palmer has made the most possible of his subject, a well worn, if not a thread bare one. It appears, however, from Dr. Palmer's report, that several failures have of late been made in the presentation of this subject, and that a hiatus of some four years elapses in the annual summary of medical publications contemplated by the rule under which this report is prepared. Dr. Palmer has declined the assumption of the almost Herculean labors involved in the reparation of the deficits from the neglects of former committees, and contents himself, "with a discussion of the general character of the periodical medical publications of the United States, referring to particulars only so far as may be necessary to illustrate general views."

The committee express their sense of embarrassment in the discussion of the subject confided to their charge, from the ability in which all the subjects appertaining to it have been discussed by former committees, and from being necessarily obliged to follow in the footsteps of some of the most illustrious of our American authors.

The committee express their conviction of the constantly improving character of our periodical medical literature, and regard our thirty medical periodicals, scattered as they are all over our country, and many having little more than a mere local circulation or name, despite the meagre returns which they pay their editors and publishers, as of positive advantage to the profession at large, from the encouragement which they give to inexperienced and modest writers who might be deterred from contributing to a journal published at a distant point abounding in a surplus of contributions and able to ignore writers unknown to fame. These local journals encourage and foster talent, and develope many gems of truth, which if it were not for the encouragement thus afforded them, would be lost to the profession from the timidity of the writers. Encouraged by notice, and perfected by practice, very many are enabled to take first rank among our medical contributors, and achieve a lasting renown. They speak, moreover, in commendation of the general style of these communications, and have a kind word to to say in reference to the taste and discrimination displayed in making selections for the eclectic department.

As a journalist, Dr. Palmer has learned from experience, many of the difficulties which practically beset the subjects confided to his charge, and states most distinctly what they are in the way to literary perfection in our medical periodical literature.

The second division or portion of the report embraces notices of "Original American Medical Publications."

This portion is occupied with distinctive notices of the more important American publications of the year, with a sort of running commentary upon the value and character of each publication.

We of course shall make no effort to reproduce this catalogue. The terms of commendation in which certain performances are referred to, might, it is true, be criticised, but we feel little disposition to be censorious in view of the earnest zeal in which the reporter has discharged his duties, and from the fact that he has not the advantage of the shield of *incog.* usually afforded reviewers when they desire to sit in judgment upon the performances of others and pass sentences of condemnation upon their labors. He labors to a disadvantage in this respect, and we would not avail ourselves of it to add zest to this notice.

The third and last division of the committee is devoted to "such measures as may be deemed advisable for encouraging and maintaining a national literature of our own."

Under this head, as usual, are discussed, or rather, are repeated, the arguments which are relied upon to point out the deficiencies in our American medical literature, and the causes, such as deficiency of early training, and want of originality of observation and a high standard of excellence upon the part of authors, which weigh down and hinder the profession in this country from assuming a front and prominent rank among medical writers, and contributors to the advancement of our science.

Passing over a page or two given to rhetorical flourishes, the greater portion of the report is occupied in the discussion of subjects springing from the consideration of the question of *the international copyright law.*

The reporter is very evidently opposed to any law, or plan which will embarrass the republication of foreign works in this country. He regards it of advantage to the profession at large, to avail themselves of all the learning of the old world, and to reproduce upon this side of the Atlantic, at a cost less than the original publication, any work entitled to consideration. He thinks no person would be deterred from buying an American work because he had one of a foreign author upon the same subject.

In conclusion, we must be permitted to say, that we regard it an evidence of exceeding bad taste to engraft in a report of this kind, a puff, which amounts to little less than an advertisement, of a practice which prevails "in at least one medical college," requiring students in medicine to exercise themselves in composition, and submit their performances to the criticism of their teachers. There is no difficulty in determining "the institution referred to." If they have adopted this among other methods of instructing and improving and perfecting their students, it is all very well and deserving of commendation; but one scarcely looks for an advertisement of "the institution referred to" in such a report, or the announcement that the crude medical papers of the student, or even his thesis, should be regarded or discussed in a report on medical literature. Schoolboy performances pass everywhere for what they are worth, and never bring a high price in the market. In the attempt to slip in covertly a puff of his school, we cannot help thinking the reporter has degraded his subject. Lessons in good taste and literary propriety, might, we think, be reflected back from the seat of the lecture room to the desk of the teacher in this case.

*The Report of the Special Committee on Medical Education,* By Jas. R. Wood, M. D., of New York, follows. This is stereotyped subject, number two.

It has the merits of brevity at least; eleven pages contain the report.

Dr. Wood, while acknowledging the importance of the subject in reference to the well being and influence of the profession, recognizes the little real good which has grown out of the constant agitation of this subject by the association. The subject had been so often reported upon, that it seemed a work of supererogation to add anything to the labors of previous committees.

The report of Dr. Wood is a tersely written paper upon the means of improving a preliminary medical education, and perfecting the medical student in whatever has reference to his future success in his profession. Not a line is lost in giving scope to any rhetorical flourishes, but the whole is directed to the means of improving and elevating the standard of medical education, as he has conceived it to be susceptible of improvement.

The most prominent point in the report undoubtedly is the prominence which is given to clinical, or hospital instruction. The advantages of such teaching to the student is strongly urged, and we presume there are very few who will be found to dissent from the position thus taken. Primary medical schools are also recommended.

We have spoken of the subjects of the last two reports as stereotyped. With all due deference to the high powers that be, and reign in, if they do not control the association, we would suggest whether it would not be to the interest of all concerned, to drop for a while subjects of report and discussion which result in so little which is valuable, or practicable.

Medical literature is beyond the control of legislation. Every man will buy books according to his own taste or fancy. American authors will supplant foreign ones when they present works equally as good. Foreign publications will cease to find such rapid sale in our markets, when American physicians ambitious of the reputation of authorship, without the qualifications to write a book, will cease to tack their names, kite-tail fashion, to the title of some foreign author, and appear in all the glories of an Editor.

We go in for anything to the fullest extent, which will encourage American talent, but you cannot force these things by legislative enactments. Let American authors depend less upon foreign aid and support, and if they write and publish less than they do now, let them when they write and publish, give us something worthy of our nationality, and we will guarantee a recognition worthy of their labors, upon the part of the profession of our land. Our American medical literature is cursed by the system of American *editorship*, but it is to the interest of American publishers to furnish us with an edition disfigured and diluted by annotations, rather than submit to the introduction, by importation and sale, of the original work, or to do what they might do in the absence of any international copyright law, give us a republication of the work as it originally came from the hands of its author. What moral right any man has to pervert the original text of an author by the addition of notes and opinions of his own, controverting as far as is in his power, the very objects probably the author had in his labors of authorship, while he steals the reputation of the writer in whose plumes he parades before the public, is a question which we have never been able to reconcile to our minds, perhaps owing to the narrow scope of our intellectual vision, which we confess, is fenced in by a recognition upon the part of conscience, (or of so much of this as we have left after the rough contact and abrasions of the world a consequence of our few years of professional service,) of the right of the *Author* to enjoy his opinions unmolested by the additions of an *Editor*. If "Mr. Editor" has anything worth imparting to the American public, let him give it to us in some manner more in keeping with our and his reputation, as an American author.

And so in reference to the subject of medical education. What practical advantage is to be gained by eternally reporting upon the subjects. The matter has been before the association since the day of its organization. How much has really resulted from the discussion of the subject?

Scarcely any two men agree upon the details involved in this question. There are innumerable local and side issues which control the judgments of those having the greatest interests in the settlement of the many points involved in the mere proposition, "Medical Education," an indefinite term, but one which from its popular and frequent use, implies imperfections in the present methods, a certainty of improvement, and a possibility of uniformity of practice which would beget a high and national standard of excellence.

There is no uniformity in our State laws in reference to medical matters. No State will disfranchise any of the members of the profession recognized by its laws, to comply with the requirements of another State, or to meet the visionary ideas of some reformer. The social condition of our States differ so much, that what may be possible to be accomplished in one portion of the Union, may be impracticable, if not impossible, in another. How is it possible to bend or to warp our profession to one fixed, uniform and rigid standard of qualifications and excellencies, under such circumstances?

Let us not be understood as depreciating medical improvements in any degree, nor the elevation of a high standard of excellence which it is the duty of every man to make the effort at least, to attain, and failing to put forth such effort, should rest under the curse of his brethren for his indolence. EXCELSIOR should be the motto of every medical man, as it is the proud motto of our proud State. What we deprecate is the Quixotic labors of inconsiderate reformers. They can more easily destroy than build up.

Why not for a few years at least, dismiss this subject from the annual reports of the association? We now simply get the crude notions of each reporter at each annual session, for each would be derilect in duty did he fail to comply with the obligation implied in his appointment, and we consequently have report after report. Probably all that can be accomplished for the present has been accomplished. Wait a few years for farther progress. In the mean time the "old fogies" will have died, or lost their influence; the younger generation will have learned what the wants of the community are, and the frequency of their intercourse at the meetings of the association will result in such a parity of views and sentiments upon the subject as will result eventually in the establishment of an uniformity much nearer to perfection than can be established, or hoped for now. It is impossible for from three to five hundred men to meet yearly, and especially if they will come together unbiassed by any preconceived project or hobby, without arriving in the course of time from the free interchange of opinions and sentiments to some definite results upon the subjects involved in the question under notice. Reports and resolutions at the present day promise nothing.

*A Report on Spontaneous Umbilical Hæmorrhage of the Newly-Born*, By J. Foster Jenkins, M. D., follows, and is really the first paper of a scientific character.

This report, which we have read with no little interest, would seem to entirely exhaust the subject. The accident itself seems to be comparatively a rare one, for according to the evidence collected by Dr. Jenkins, practitioners of a quarter of a century have not met with an instance, and at the Foundling Hospital, at Paris, but a single case occurred in between 9000 and 10,000 children admitted in two years, preceding January, 1853. The records of other hospitals abroad, as well as in this country, testify to its comparative rarity. Until quite recently, works upon infantile diseases have given it but slight attention. At the same time, we presume, no medical man has been free from anxiety, or indifferent to the dangers attending this difficulty, when called upon to take charge of a case.

Dr. Jenkins has prosecuted with remarkable industry and zeal, his investigations in the attempt to elucidate the pathology of this infantile disease, if disease it be, for his conclusions would seem to point to the inference that the cause depended upon some congenital malformation or imperfect action of the hepatic organs. His laborious research has collected 178 cases, which he has tabulated and submitted to careful comparison and analysis.

His conclusions are, from such study, that they point to the existence of two varieties of the form of umbilical hæmorrhage under discussion.

"First, And most common, that depending on a depraved condition of the blood, the spanæmia resulting sometimes from jaundice, through malformation, or deranged function of the liver, sometimes from an inherited scrofulous or syphilitic taint, and probably not unfrequently from privation and despondency in the mother during gestation, or during the same period an excessive use of alkalies or diluent fluids.

"Second. Independently of any dyscrasia of the blood, umbilical hæmorrhage seems to arise by reason of an usual patency of the umbilical vessels, in otherwise apparently healthy children.

"These two conditions of the hæmorrhagic flow are doubtless often associated, but that the second exists sometimes exclusively, may not be questioned."

Male children, according to authors and reporters, seem to be more liable to this accident than female. Some dependence upon a hereditary hæmorrhagic diathesis seems to be traced, but the sequence is not sufficiently uniform to establish any law.

The prognosis is fearfully grave. Five-sixths (83.7 per cent.) of those attacked succumbed to the disease, or its sequelæ, or 149 out of the 178 cases collected in the report.

The report evinces commendable industry upon the part of the reporter, but we fear that the practical advantages are scarcely commensurate with the labor he has bestowed upon the subject. The almost certain fatal termination of the accident, renders of but little avail speculations in pathology. His suggestions for treatment are such as are well known to the profession from other sources, or would be suggested to the mind of any intelligent practitioner.

*The Report on Influence of Marriages of Consanguinity Upon Offspring*, By S. M. Bemiss, M. D., of Louisville, is another lengthy paper of tabulated figures, and a monument of patient labor upon the part of the reporter. He has had a field comparatively new to labor in, for although a sort of general assent has been given to the fact that marriages of relations were disastrous in their consequences to the offspring of such unions, but few figures have been heretofore collected to solve the problem.

The conclusions of the reporter are drawn from near nine hundred such marriages, and he has collected for comparison one hundred and twenty-five observations where the parties were in no way related to each other. The assiduity with which those researches have been prosecuted, is apparent. Dr. Bemiss gives full credit to those who have assisted him, and the instances where other parties who were engaged in the same investigations assisted him to what they had accomplished, and resigned the anticipated credit properly accruing to such labors, in favor of the committee appointed upon the behalf of the association, he pays a fitting and graceful compliment.

The points sought to be elucidated by this report, are worked out in almost innumerable tables, and with a painstaking labor worthy of all commendation. Dr. Bemiss proves himself to be a statistician of the first order. We fear that his labors will scarcely be appreciated by the many careless readers of the volume, who will scarcely stop to examine with the study which their perusal requires, the details of so many pages of figures. If it be desirable that public attention should be called to this subject, and the impression fixed upon their minds, that such unions are disastrous to their offsprings, it is much to be regretted that some more effectual means than the simple publication of the results of these inquiries in the volumes of the Transactions of the Association could not be devised. So far as the public are concerned they will be lost to them. The report will furnish part of the lore of the physician, and supply materials for the vital statistician, but we fear will be so inaccessible to the community, that the objects of all this labor will be but very imperfectly accomplished in the correction of the evil which they point out.

It is scarcely to be expected that we can reproduce in this notice, the points worked out in the ninety pages of tables. We must refer those who are curious upon the subject, to the careful perusal of the report itself.

It will, perhaps, be sufficient to state, that the conclusions drawn from the facts presented in this report, are of a startling character. The mental and physical degeneracies manifested in the offspring of blood relations as exhibited in the statistics gathered in this report are such as to attract the most earnest attention, and if future investigations upon the subject sustain the conclusions drawn from the figures before us, it may well be a question whether it should not be taken under legislative control, if the moral sentiments of the community cannot be educated up to a point, to pay voluntarily a deference to the laws which nature seems to have laid dawn in reference to this matter, and a violation of which she seems to so fearfully punish A broad field is opened for investigation and study. The results are of most manifest importance to the well being and happiness of the race. The subject is a delicate as well as an important one, and requires careful handling, as it comes in contact with so many social interests of society. Dry statistics and figures may, however, prove too much for sentiment, and stand guard over the avenues of affection, and dictate whom one may and may not wed. It would seem as if all of the interests of life were being reduced to a mathematical problem, and the square and compass were to be applied to the minutest of our relations to this world.

*Report on the Functions of the Cerebellum,* By E. Andrews, M. D., of Chicago, follows.

We think the reporter has been exceedingly unfortunate in stating the especial object or intention of this paper. It bears upon its face the evidence of labor; and the illustrations, drawn from the results, as many of them are, of his own dissections, display the student. But we fear many a reader will be puzzled to discover the especial point sought to be elucidated. There is too much ambiguity in the annunciation of the particular objects of the report, to make its perusal at all pleasant, or profitable. For ourselves we confess to the labor of more than one perusal, in search of the particular point, or points, it is designed to prove.

That the functions of the cerebellum are supposed to be exerted in controlling muscular movements, is, we believe, among the admitted physiological actions of this portion of the cerebral mass, if not demonstrated. Without spending time to hunt up authorities, we would simply direct the curious to no further search than that of simply turning to the pages of "Todd and Bowman's Physiological Anatomy," upon the subject of the *functions of the cerebellum.*

The specific object of the report seems to be to sustain the two following propositions:

"1. In the warm blooded animals, the median lobe, or vermiform process of the cerebellum varies in size directly as the bulk and power of the anterior group of muscles.

"2. The lateral lobes vary in like manner, as the power of the posterior group of muscles; subject, however, to certain variations hereafter to be mentioned."

The author may have regarded the above announcement sufficient for the purpose, but we scarcely think the reader will coincide in his opinion of the perspicuity of style, and that his method of treating his subject amounts to a demonstration, physiological, or otherwise.

*Report on the Treatment Best Adapted to Each Variety of Cataract,* By Mark Stephenson, M. D., New York, is a well written paper upon the different forms of operation adapted to this distressing infirmity. His views upon what is commonly called preparatory treatment for an operation, and of the discretion to be used in meeting subsequent inflammation are exceeding judicious, and will commend themselves to the reader. The peculiar advantages of each form of operation are also well described. But at the same time, the paper can lay no claim to any originality in theory or practice, and the sum of our knowledge upon ophthalmic surgery can scarcely be said to be increased by the labors of the reporter.

*The Report on the Law of Registration of Births, Marriages, and Deaths,* By EDWARD JARVIS, M. D., of Dorchester, Mass., states that the committee was appointed at a former session of the association, of members dispersed throughout all the States of the Union for the purpose of bringing this subject to the notice of their respective legislatures. But there had been no meeting of the committee, nor any concert of action of any kind between them in reference to the matter.

Dr. Jarvis recapitulates in this report the steps Massachusetts has taken for the collection of this class of statistics, under the sanction of legislative enactments. Since 1842, the laws in reference to registration in these several departments of vital statistics have been kept in vigorous and successful operation. Reports have been annually published since that date.

These reports are probably the most reliable and valuable of any,published in the United States, unless we except those of Rhode Island, which although of much more recent date in the undertaking, and from a smaller territory, are crowding hard upon the returns of its sister State for minuteness and accuracy.

The report is closed by the additions of the Nosology prepared by Dr. Jarvis for the Mortality Statistics of the United States for 1850, published under the supervision of Mr. De Bow; and of the classification of diseases arranged by Dr. Wm. Farr, of London.

It perhaps may not be out of place to refer in this connection to some action taken by the Association in reference to procuring a proper national recognition of the importance of the subject involved under the head of vital statistics, and giving the full influence of the Association to the support of the same.

On the motion of Dr. W. L. Sutton, it was

"*Resolved,* That a committee of three physicians of the District of Columbia, be appointed, whose duty it shall be to wait upon the Census Bureau and impress upon the proper officer the great interest which this Association feels in the proper collection of the details contemplated by the laws for taking the census of 1860; and especially that portion which relates to vital statistics; and to urge the importance of securing, at an early day, the services of a physician versed in vital statistics, to assist in constructing the schedules, and in superintending this branch of the census."

It was also upon the motion of Dr. Sutton, further

"*Resolved*, That a committee of five be appointed, whose duty it shall be to prepare a plan for an uniform system of registration of births, marriages, and deaths; including the nomenclature and classification of causes of death, to be recommended to the several States for adoption. And that the report by Dr. Jarvis, of Mass., to this meeting, be submitted to them, during the remainder of this session."

A committee of medical gentlemen well known to the profession for their labors in the department of vital statistics, was appointed, but we do not discover that they made any report during the session of the Association. It was hardly to be expected that they would be able to do anything of the kind, in the short period of the session. The gentlemen appointed, are, however, too much devoted to this branch of medical statistics to suppose that they will allow the subject to sleep a final sleep in their hands.

*Report on Stomatitis Materna.* By D. L. M'Gugin, M. D., of Keokuk, Iowa.

This subject has afforded a prolific source for contributions to medical journals, within a few years past.

Dr. M'Gugin criticizes in the opening of his report the terms employed to give a name to this disease, and complains of the difficulties of according it a proper place in a nosological list. He denies that the disease is of recent origin, notwithstanding practitioners and authors of eminence testify that they have never met with a case in their practice. He concludes it to be as old as the day of Hippocrates, and finds evidence in his writing of its existence. The history, topography, and bibliography of the disease as traced by the reporter, is exceedingly interesting and will well repay perusal.

From the observations of the reporter, this disease would seem to be endemic in its character, and much more frequent in the western than in the eastern sections of our land. This is certainly a point worthy of the consideration of the profession, and well authenticated observations and reliable deductions are necessary to establish the fact whether so distressing a complication of the periods of gestation and lactation as it frequently is, to say nothing of its occasional fatality, is depending upon local causes. It is difficult to conceive that such a dependence exists. It was our misfortune the past summer, to lose a patient with this disease, but we can recall to mind no local causes likely to have produced the disease; and at the same time we can testify that it is scarcely conceivable to one who has never witnessed a case, how anæmic a nursing woman may become, before death releases the sufferer from her agony.

To turn to the practical part of the report, that which has reference to treatment, we do not perceive that our stock of knowledge is much increased. Hydriodate and chlorate of potassa, are highly spoken of, and the latter has been found most efficacious. Benefit has been found also from the use of the hypophosphite of soda in syrup, subnitrate of bismuth, and malt liquors. Exercise in the open air is enjoined as indispensable.

While the report, perhaps, adds nothing to our knowledge of the etiology and pathology of the disease, we commend it to the careful perusal of every practitioner, as giving a very perfect description of the symptoms of the disease, and as presenting about all that may be regarded as its literature.

*Report on the True Position and Value of Operative Surgery as a Therapeutic Agent*, By J. B. Flint, M. D., of Louisville, is, we think, an exceedingly unfortunate title for a *paper* containing much practical and sound good sense. We think very few will be able to discover any applicability of the title to the contents of his communication.

Instead of being what may be properly called a *report*, it is a *paper*, or an *essay*, the intention of which is to deprecate the passion which so many practitioners, and young ones especially, have for achieving sudden renown and reputation by the performance of some formidable and bloody, and often needless and useless, surgical operation. The *eclat* which attends such performances over the more quiet prescription of the physician is severely criticised and satirized. In this light the contribution of Prof. Flint is entitled to the careful study of all those who are ambitious to *carve* their way to medical, or rather, surgical fame. The contribution is strongly inculcative of what is known in this region as *conservative surgery*.

A point made in the paper is the disparity in the fees willingly accorded by the patient for a valuable and effectual medical prescription, and for a surgical operation, perhaps useless, but which impresses itself upon patient and observer, because blood follows in the track of the knife; and because public estimate has accorded to surgery a position from the strong impression it makes upon the senses from the sight of the sufferings and blood of the patient, disproportionate to the more positive relief often afforded by some quiet prescription, or unseen and bloodless manipulation.

(Want of space in this number of the Journal, and uncontrolable circumstances, which for the present put an end to all use of the pen, force us to close thus abruptly our notice of this volume. At some future time the remaining reports may be taken up and examined. Some of the papers are so lengthy that they will admit of separate notices.) J. M. N.

